# Special section on honey bee health and disease

**DOI:** 10.1177/10406387231202959

**Published:** 2023-10-10

**Authors:** Marie-Odile Benoit-Biancamano

**Affiliations:** Groupe de recherche sur les maladies infectieuses en production animale (GREMIP), Centre de diagnostic vétérinaire de l’Université de Montréal (CDVUM), Département de pathologie et microbiologie, Faculté de médecine vétérinaire, Université de Montréal, Saint-Hyacinthe, Québec, Canada


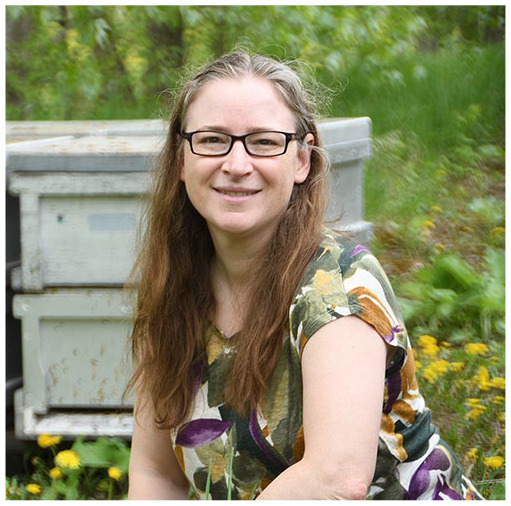


Honey bees are important production animals that play a significant role in the agricultural economy as well as in the human and animal food supply. Not only do honey bees provide honey, but they are also key to commercial plant pollination, which improves crop yield for a large variety of marketable plant species.^10^ Like other production animals, bees are susceptible to a number of pathogens and toxicants; colony losses over the last several decades have increased the public’s awareness of honey bee health, and gained the interest of researchers with a wide array of expertise.^7,9^ We invited submissions for our JVDI special section to compile some of the most recent advances in the development of diagnostic tools and understanding of diseases specific to honey bees, which we hope will be helpful to diagnosticians.

Although most veterinarians are not accustomed to working with production insects, veterinarians can play an important role in maintaining bee health.^15^ The pathogens encountered in honey bees include not only a large number of viruses, but also bacteria, parasites, and fungi.^1,4,14^ *Varroa destructor*, the most pathogenic bee parasite, plays a central role in transmitting several viruses, and hence deserves specific scientific attention.^12,13^

An increasing number of veterinary diagnostic laboratories offer services to apiarists and bee veterinarians. Most of the usual laboratory techniques (e.g., PCR, bacterial culture, histopathology) can be adapted for honey bees, although some require adjustments specific to this species.^5,6,11^

The scientific and general population have expressed concerns about human, livestock, and pollinator exposure to potentially harmful pesticides, especially neonicotinoids.^3,8^ For this reason, much research has aimed at detecting residues and studying their effects in different species,^2,3,8^ and the use of several pesticides has been prohibited in various countries over the last decade, particularly in the European Union.

Researchers and diagnostic laboratories can play a central role in elucidating the pathogenesis of honey bee diseases and in providing tools for apiarists and field veterinarians who intervene to maintain honey bee colony health. With this renewed impetus, we look forward to continued discoveries in the field of pathology and diagnosis of honey bee diseases.
